# Airflow limitation or static hyperinflation: which is more closely related to dyspnea with activities of daily living in patients with COPD?

**DOI:** 10.1186/1465-9921-12-135

**Published:** 2011-10-11

**Authors:** Koichi Nishimura, Maya Yasui, Takashi Nishimura, Toru Oga

**Affiliations:** 1 Department of Respiratory Medicine, Rakuwakai Otowa Hospital, Kyoto, Japan; 2 Kyoto-Katsura Hospital, Kyoto, Japan; 3 Department of Respiratory Care and Sleep Control Medicine, Graduate School of Medicine, Kyoto University, Kyoto, Japan

**Keywords:** Chronic obstructive pulmonary disease, Airflow limitation, Hyperinflation, Dyspnea, Baseline Dyspnea Index

## Abstract

**Background:**

Dyspnea while performing the activities of daily living has been suggested to be a better measurement than peak dyspnea during exercise. Furthermore, the inspiratory capacity (IC) has been shown to be more closely related to exercise tolerance and dyspnea than the FEV_1_, because dynamic hyperinflation is the main cause of shortness of breath in patients with COPD. However, breathlessness during exercise is measured in most studies to evaluate this relationship.

**Purpose:**

To evaluate the correlation between breathlessness during daily activities and airflow limitation or static hyperinflation in COPD.

**Methods:**

We examined 167 consecutive outpatients with stable COPD. The Baseline Dyspnea Index (BDI) was used to evaluate dyspnea with activities of daily living. The relationship between the BDI score and the clinical measurements of pulmonary function was then investigated.

**Results:**

The Spearman rank correlation coefficients (Rs) between the BDI score and the FEV_1_(L), FEV_1_(%pred) and FEV_1_/FVC were 0.60, 0.56 and 0.56, respectively. On the other hand, the BDI score also correlated with the IC, IC/predicted total lung capacity (TLC) and IC/TLC (Rs = 0.45, 0.46 and 0.47, respectively). Although all of the relationships studied were strongly correlated, the correlation coefficients were better between dyspnea and airflow limitation than between dyspnea and static hyperinflation. In stepwise multiple regression analyses, the BDI score was most significantly explained by the FEV_1 _(R^2 ^= 26.2%) and the diffusion capacity for carbon monoxide (R^2 ^= 14.4%) (Cumulative R^2 ^= 40.6%). Static hyperinflation was not a significant factor for clinical dyspnea on the stepwise multiple regression analysis.

**Conclusion:**

Both static hyperinflation and airflow limitation contributed greatly to dyspnea in COPD patients.

## Background

Dyspnea is multifactorial, but static lung hyperinflation and its increase during exercise (dynamic hyperinflation) is believed to be the most important in subjects with chronic obstructive pulmonary disease (COPD) [1-3]. It has been reported that indices related to hyperinflation, such as the inspiratory capacity (IC), are more closely related to exercise tolerance and dyspnea than the forced expiratory volume in 1 second (FEV_1_) or forced vital capacity (FVC) [4-8]. The Borg scale is frequently used during exercise as a marker of laboratory dyspnea in physiological investigations to evaluate this relationship. Furthermore, Casanova and colleagues proved that static lung hyperinflation estimated by the inspiratory capacity-to-total lung capacity (IC/TLC) ratio is a predictor of all-cause and respiratory mortality in patients with COPD, independent of the FEV_1 _[9].

The outcome measurements for dyspnea can be broadly divided into those that assess breathlessness during exercise (laboratory dyspnea), and those that assess overall breathlessness during daily activities (clinical dyspnea). Using factor analysis, Hajiro et al. [10] reported that various clinical dyspnea ratings were virtually identical for evaluating dyspnea in COPD patients. On the other hand, dyspnea at the end of maximal exercise may provide a different type of information regarding dyspnea [10]. It has also been reported that dyspnea during daily activities was more significantly correlated with objective and subjective measurements of COPD than dyspnea at the end of exercise, and that the former was more predictive of mortality [11]. Therefore, dyspnea while performing the activities of daily living is considered to be a better measurement for evaluating the disease severity of COPD than peak dyspnea during exercise.

We hypothesized that static hyperinflation may be more closely related to clinical dyspnea than laboratory dyspnea, since there is a close relationship between the IC and exercise performance and dyspnea in COPD. The purpose of this observational study was to evaluate the correlation between breathlessness during daily activities measured using the Baseline Dyspnea Index (BDI) and airflow limitation or static hyperinflation in COPD.

## Methods

A total of 167 consecutive patients with stable COPD defined as a FEV_1_/FVC of less than 0.7 for all measurements made during the previous 6 months were recruited at the outpatient clinic of the Respiratory division of Kyoto-Katsura Hospital. The entry criteria included: (1) a diagnosis of COPD and an age over 40 years; (2) a self-reported current or former smoker; (3) regular attendance at our clinic for more than 6 months to avoid substantial changes in subjective parameters brought about by new medical interventions; and (4) no changes in the treatment regimen for more than 4 weeks. Patients with any history suggestive of asthma, a never-smoker, an exacerbation of their COPD over the preceding 6 weeks, previous inflammatory changes revealed on chest radiographs that could influence pulmonary function (for example, a previous thoracoplasty or tuberculous sequelae), or any other illnesses, were excluded. All eligible patients underwent the following examinations on the same day. None of the results from the 167 patients in the present study have been published elsewhere. Informed consent was obtained from all participants.

On the evaluation day, the patients completed their pulmonary function tests, arterial blood gas (ABG) analyses, blood investigation, chest X-rays and dyspnea measurements. The patients were requested to stop using tiotropium bromide for 24 hours before, and were also asked to discontinue the use of other inhaled bronchodilators for at least 12 hours before the assessment. According to the method described by the ATS/ERS Task Force in 2005 [12], three acceptable spirometric flow-volume curves were recorded with the patient sitting using a calibrated 2.0-L syringe before every measurement. The largest FEV_1 _and the largest FVC among three maneuvers were then analyzed. The predicted values for the FEV_1 _and vital capacity (VC) were calculated according to the proposal from the Japan Society of Chest Diseases [13]. The residual volume (RV) was measured by the closed-circuit helium method, and the diffusion capacity for carbon monoxide (DLco) was measured using the single-breath technique (CHESTAC-65 V; Chest, Tokyo, Japan). Chest radiographs were obtained in all patients. ABG analyses were also performed. In cases associated with long-term domiciliary oxygen therapy, the arterial blood was obtained while breathing the predetermined oxygen therapy. Blood was collected to measure the levels of plasma brain natriuretic peptide (BNP) by a chemiluminescent enzyme immunoassay [14].

To assess dyspnea, the Japanese version of the Baseline Dyspnea Index (BDI) was used [10,15,16], which has been previously validated. The BDI recognizes five grades for each of the following categories: functional impairment, magnitude of task and magnitude of effort, with higher scores indicating more severe dyspnea. The original Japanese version of the BDI/TDI was completed and the first two studies for validation were published in 1998 [10,16]. The newer Japanese version of the BDI/TDI was subsequently developed and replaced the older version in 2008. However, the former Japanese version of the BDI was used in the present study.

### Statistical Analysis

All results are expressed as means ± SD. The relationship between two sets of data was analysed by both Spearman's rank correlation and by Pearson's correlation tests. Multiple regression analysis was performed to determine the association of the various variables with the BDI scores. The independent variables analysed were: age (years), smoking (pack-years), body mass index (BMI) (kg/m^2^), FEV_1 _(L), IC/predicted TLC, DL_CO_ (mL/min/mmHg), PaO_2 _(mmHg) and blood BNP levels (pg/mL). The FEV_1 _and IC/predicted TLC were selected as indices for airflow limitation and static hyperinflation, respectively. Multiple linear regressions were obtained by the standard, forward and backward stepwise methods. A p value of less than 0.05 was considered to be statistically significant.

## Results

**Figure 1:**
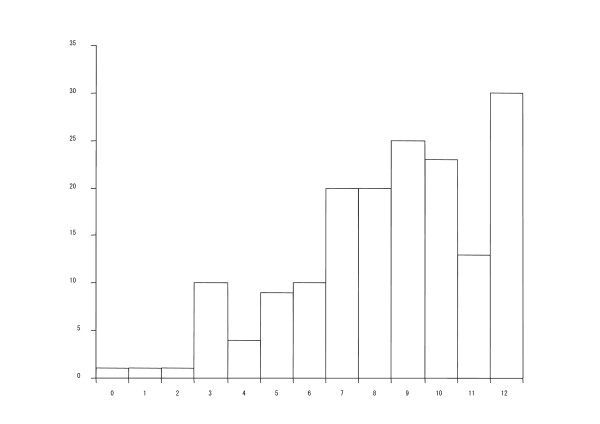
**Frequency distribution histograms of the BDI scores**. Lower scores indicate more severe dyspnea.

A total of 167 consecutive patients (147 males) were studied at the outpatient clinic between September 2007 and September 2008. Their demographic details as well as pulmonary function test data are shown in Table 1. The average age and FEV_1 _were 71.6 ± 8.7 years and 1.52 ± 0.72 L, since the patient group included cases with mild to severe airflow limitation. All patients except for two were treated with inhaled bronchodilators plus high doses of inhaled corticosteroids. Six subjects were also given oral corticosteroids. Eight patients were treated with long-term oxygen therapy. Three subjects were managed with non-invasive positive pressure ventilation at home. The frequency distribution histograms of the BDI scores in the present study are shown in Figure 1. The scores are skewed towards the very mild end of the scale.

**Table 1:** Demographic details and correlations with the BDI score (Spearman's rank correlation test) in 167 subjects with stable COPD.

variable	units	mean	SD	max	min	Correlations with BDI score	
						
						Rs	p value
Age	years	71.6	8.7	90	40	-0.25	0.0014
BMI	kg/m^2^	21.1	3.2	32.2	13.3	0.17	0.0320
Cumulative Smoking	pack-years	71	42	268	4	-0.23	0.0033
VC	Liters	3.21	0.91	5.31	0.97	0.45	< 0.0001
VC	% pred	102.6	22.8	156.2	38.6	0.48	< 0.0001
FVC	Liters	3.05	0.89	5.25	0.99	0.47	< 0.0001
FVC	% pred	97.4	22.5	147.9	40.9	0.51	< 0.0001
FEV_1_	Liters	1.52	0.72	3.46	0.39	0.60	< 0.0001
FEV_1_	% pred	68.5	27.4	133.0	13.0	0.60	< 0.0001
FEV_1_/FVC	%	48.2	13.9	69.9	22.1	0.56	< 0.0001
TLC	Liters	5.65	1.15	8.49	2.86	0.24	0.0022
TLC	% pred	111.3	17.5	160.6	57.3	0.24	0.0022
IC	Liters	2.09	0.68	3.57	0.69	0.45	< 0.0001
IC/TLC	%	36.8	8.6	60.3	11.8	0.47	< 0.0001
IC/predicted TLC	%	41.1	11.7	66.5	12.2	0.46	< 0.0001
DLco	mL/min/mmHg	10.26	5.45	24.66	0.22	0.55	< 0.0001
DLco	% pred	65.0	27.3	133.7	2.5	0.53	< 0.0001
DLco/V_A_	mL/min/mmHg/L	2.35	1.16	6.52	0.03	0.52	< 0.0001
PaO_2_	mmHg	80.1	11.5	104.8	50.3	0.48	< 0.0001
PaCO_2_	mmHg	40.5	4.8	63.3	30.7	0.00	0.98
pH (arterial blood)		7.43	0.03	7.53	7.33	0.04	0.64
BNP	pg/mL	46.5	78.8	521.0	3.0	-0.23	0.0033
BDI score	(0-12)	8.5	2.8	12	0	-	-

Gender	147 Male/20 Female						
Smoking Status	29 Current/138 Former						

Table 1 shows the correlations between the BDI and 22 characteristics, and statistically significant correlations were observed between the BDI scores and 20 characteristics excluding the PaCO_2_. There was no correlation between the acid-base balance and the BDI scores. Table 2 shows simple correlations between three airflow limitation characteristics and the BDI scores, as well as between three static hyperinflation characteristics and the BDI scores. The Spearman rank correlation coefficients between the BDI score and the FEV_1 _(L), FEV_1 _(%pred) and FEV_1_/FVC were 0.60, 0.56 and 0.56, respectively. On the other hand, the BDI score was also correlated with the IC, IC/predicted TLC and IC/TLC (Rs = 0.45, 0.46 and 0.47, respectively). Although all of the relationships studied were strongly correlated, the correlation coefficients were better between dyspnea and airflow limitation than between dyspnea and static hyperinflation. These results were similar when the Pearson's correlation coefficient was used instead (Table 2).

**Table 2:** Correlations of the BDI score with airflow limitation and static hyperinflation.

	Spearman's rank correlation coefficients		Pearson's correlation coefficient	
	
	Rs	p value	R	p value
Dyspnea vs. Airflow limitation				
BDI score vs. FEV_1 _(L)	0.60	< 0.0001	0.60	< 0.0001
BDI score vs. FEV_1 _(%pred)	0.56	< 0.0001	0.57	< 0.0001
BDI score vs. FEV_1_/FVC	0.56	< 0.0001	0.57	< 0.0001

Dyspnea vs. Static Hyperinflation				
BDI score vs. IC (L)	0.45	< 0.0001	0.48	< 0.0001
BDI score vs. IC/predicted TLC	0.46	< 0.0001	0.51	< 0.0001
BDI score vs. IC/TLC	0.47	< 0.0001	0.48	< 0.0001

Stepwise multiple regression analyses were performed to identify those variables that could best predict the dyspnea assessed by the BDI score. The FEV_1 _(L) and IC/predicted TLC, the airflow limitation and static hyperinflation characteristics with the strongest simple correlations with dyspnea in Table 2, as well as six other independent variables from Table 1 were included. We found out that the airflow limitation (FEV_1_) and diffusion capacity for carbon monoxide (DLco) significantly accounted for 26.2% and 14.4% of the variance, respectively (Table 3). Since the cumulative R^2 ^was 0.406, unknown factors still contribute to the BDI score. However, static hyperinflation was not a significant factor for clinical dyspnea using stepwise multiple regression analysis. The results were the same when we analyzed the 101 subjects with moderate to very severe COPD (excluding mild COPD) (data not shown).

**Table 3:** Results of stepwise multiple regression analyses to identify those variables that best predicted dyspnea assessed by the BDI score.

	BDI score
Independent variables	
Age (years)	-
Smoking (pack-years)	-
BMI (kg/m^2^)	-
FEV_1 _(L)	0.262
IC/predicted TLC	-
DL_CO_ (mL/min/mmHg)	0.144
PaO_2 _(mmHg)	-
BNP (pg/mL)	-
Cumulative R^2^	0.406

## Discussion

The reason why patients with COPD feel subjective dyspnea is a simple question. However, answering this question is not simple, and clinicians need to understand the mechanisms responsible for dyspnea. It is widely accepted that the major limitation to exercise performance and the perception of breathlessness in COPD can be attributed to dynamic hyperinflation, although activity limitation and dyspnea in COPD is multifactorial. This has been explained by the following mechanism [1-3]. In COPD, the end-expiratory lung volume (EELV) is elevated as compared to healthy controls. During spontaneous breathing at rest in patients with expiratory flow limitations, the EELV is maintained at a level above the statically determined relaxation volume of the respiratory system. In flow-limited patients, the mechanical time-constant for lung emptying is increased in many alveolar units, but the expiratory time available during quiet breathing is often insufficient to allow the EELV to completely decline to its normal relaxation volume, and thus air trapping results. Dynamic hyperinflation occurs in flow-limited patients under the condition of increased ventilatory demand during exercise. Since the total lung capacity does not change during activity, the decrease in the IC must reflect an increase in the dynamic EELV, or the extent of dynamic hyperinflation. With the limitation of the tidal volume increase during exercise, dynamic hyperinflation results in restrictive mechanical constraints which, in the extreme, can lead to alveolar hypoventilation during exercise. In patients with COPD, breathing to higher lung volumes increases the total respiratory work, and thus potentiates the perception of breathlessness, which favors a decrease in physical activity.

The rate and magnitude of dynamic hyperinflation during exercise is generally measured in the laboratory setting by serial inspiratory capacity measurements. O'Donnell et al. reported that the exercise endurance time, Borg dyspnea ratings at the isotime near end-exercise, and IC are very reproducible indices [5], and that 500 micrograms of nebulized ipratropium bromide can improve the exercise endurance time by 32% on average. This improvement correlated best with the IC improvement, but not with the FVC or FEV_1 _improvements, and the change in the Borg dyspnea ratings at the isotime near end-exercise also correlated well with the IC improvement [6]. An increased IC means reduced resting lung hyperinflation. Using a similar mechanism, the use of tiotropium bromide, salmeterol, or a fluticasone propionate/salmeterol combination was associated with sustained reductions in lung hyperinflation at rest and during exercise. The resultant increases in inspiratory capacity permitted a greater expansion of the tidal volume, and contributed to improvements in both exercise endurance and exertional dyspnea [4,7,8].

In the present study, airflow limitation may have been a more important cause of clinical dyspnea than static hyperinflation. This clearly contradicts the above mentioned hypothesis, and the results of the laboratory exercise tests that are based upon it. Why is our result different? The first issue to consider is the different dyspnea evaluation methods used. We wanted to assess overall breathlessness during daily activities (clinical dyspnea) using the BDI score in the present study, whereas the Borg dyspnea ratings at isotime exercise has been used in most laboratory studies. Dyspnea during exercise using the Borg scale may provide a different type of information regarding dyspnea than clinical dyspnea [11]. Therefore, if the cause of COPD dyspnea is hypothesized to be dynamic hyperinflation, then it is necessary to evaluate clinical dyspnea instead of laboratory dyspnea.

Murariu et al. used a method similar to ours, and evaluated their maximal symptom-limited exercise on a cycle ergometer. Their correlation coefficients between the Wmax with the IC and FEV_1 _were 0.81 and 0.54, respectively, and a multiple regression model using the Wmax as the dependent variable revealed that the IC was the only significant contributor to the Wmax. They also reported that the FEV_1 _was not statistically significant [17]. Their study used the Wmax as the outcome, whereas we used clinical dyspnea instead. Although the methods of their analysis were similar, their comparison between airflow limitation and static hyperinflation resulted in completely different conclusions. Therefore, using clinical dyspnea as the outcome in our study probably explains the different results.

The main reason why dynamic hyperinflation can be hypothesized to be the main cause of dyspnea is the strong correlation between dynamic hyperinflation and dyspnea. Some researchers have argued against this hypothesis, since the presence of dynamic hyperinflation is not a universal finding during exercise [18]. We did not directly evaluate dynamic hyperinflation, but instead used the IC, which is the index for static hyperinflation. The IC may reflect dynamic hyperinflation inaccurately. Nevertheless, in the study conducted by O'Donnell et al., the correlation between the magnitude of the changes in the IC and Borg scores was strong, and they concluded that this explained why dynamic hyperinflation was causing dyspnea. However, correlations in cross-sectional studies and longitudinal studies do not necessarily match, and a statistical approach such as correlation coefficients may not resolve this issue. Airflow limitation causes dynamic hyperinflation, and hence airflow limitation, dynamic hyperinflation and dyspnea may be considered as the top of a pyramid, and it may not be necessary to consider them in a linear, causal relationship.

In the present study, airflow limitation explained only 26% of the BDI score, and airflow limitation plus the diffusing capacity explained an accumulative 41% of the BDI score. In the literature, it is thought that dyspnea measures are moderately correlated with pulmonary function, psychological function, and walking tests [19]. For example, a simple correlation between the BDI score and FEV_1 _has been reported to be statistically significant, with a correlation coefficient of 0.22-0.58 [10,19-21]. Although as per pulmonary function, the FEV_1 _and FVC are often evaluated for a correlation with clinical dyspnea, the correlations between the FEV_1_, static hyperinflation and clinical dyspnea have not been evaluated simultaneously. In addition, to our knowledge, this is the first study which proved that the diffusing capacity was a significant contributor to clinical dyspnea. This may indicate that emphysema-predominant subjects with COPD are conscious of stronger dyspnea. Our results obtained from the stepwise multiple regression analyses also indicate that there are other unmeasured factors that explain clinical dyspnea. Wijkstra et al. [22] reported that the transfer factor for carbon monoxide (T_LCO_) was strongly correlated with the six minute walking test and with the maximal work load, and that backward linear regression analysis selected the T_LCO _and peak esophageal pressure during a maximal semistatic maneuver as the most significant determinants for exercise performance. However, although they discussed the mechanism of correlation between the T_LCO _and exercise capacity, their cause-effect relationship is still unknown. Similarly, the mechanism of correlation between the diffusing capacity and clinical dyspnea is also unknown

There are also important considerations in the clinical practice setting. A common misunderstanding is that hypoxemia is causing dyspnea, and proper oxygen administration alone is enough. We want to emphasize that oxygen administration to alleviate dyspnea in COPD patients whose PaO_2 _is over 60 mmHg is the wrong treatment.

Since some researchers understand that COPD is a systemic disease, we should consider that other many factors possibly related to dyspnea. Since depression and anxiety are frequent in subjects with COPD, they have been investigated for their role in clinical dyspnea [19]. Unfortunately, a psychological assessment was not included in the present study.

We measured the BNP levels to investigate whether heart failure can play a role in dyspnea in COPD patients. It has been reported that BNP can be used to differentiate heart failure from respiratory diseases, including COPD, in patients with dyspnea [23]. Furthermore, COPD patients were reported to have higher levels of BNP as compared to controls [24]. Although the Spearman rank correlation test revealed a significant correlation between BNP levels and dyspnea, the stepwise multiple regression analysis did not. This does not explain what elevated BNP levels in subjects with COPD mean clinically, but the magnitude of this elevation may depend on the disease severity instead of dyspnea.

Some limitations of the present study should be mentioned. Most of the issues are related to the study design. First, this study is based just on correlation analysis, which is not the best way to detect the cause of a phenomenon. Second, although stepwise multiple regression analyses were performed to compare the relative contributions between airflow limitation and static hyperinflation on clinical dyspnea, over half of the contributory factors are still unknown. Third, we analyzed the FEV_1 _(L), FEV_1 _(%pred) and FEV_1_/FVC as for airflow limitation. Although the FEV_1 _is very popular, it may be an older index of flow limitation. Other methods, including the tidal volume over the envelope in the flow-volume loop or the negative expiratory pressure during tidal breathing, should be compared against any measurements of clinical or laboratory dyspnea. The present study was also limited by the small number of participants and distinct male preponderance of the subjects. Although the latter is typically observed in subjects with COPD in Japan, generalization of these results to women with COPD may be uncertain.

## Conclusion

Both static hyperinflation and airflow limitation contributed greatly to dyspnea in COPD patients. Our conclusion does not support the hypothesis that the perception of breathlessness in COPD is attributable to static hyperinflation. One possible reason for this inconsistent conclusion may be that different types of dyspnea (clinical dyspnea vs. laboratory dyspnea) have been assessed in previous investigations.

## Abbreviations

COPD: chronic obstructive pulmonary disease; IC: inspiratory capacity; FEV_1_: forced expiratory volume in 1 second; FVC: forced vital capacity; TLC: total lung capacity; BDI: Baseline Dyspnea Index; ABG: arterial blood gas; RV: residual volume; DLco: diffusion capacity for carbon monoxide; BNP: brain natriuretic peptide; BMI: body mass index; EELV: end-expiratory lung volume; TLco: transfer factor for carbon monoxide

## Competing interests

KN has received lecture fees from Boehringer-Ingelheim and GlaxoSmith-Kline, but not in relation to the topic of the current manuscript. The other authors declare that they have no competing interests.

## Authors' contributions

KN was the physician responsible for all participants, developed the study design, and prepared the manuscript. MY and TN participated in the data collection and care for the participants. TO performed the statistical analysis. All authors read and approved the final manuscript.
